# Enhanced osteogenic activity of titania-modified zirconia implant by ultraviolet irradiation

**DOI:** 10.3389/fbioe.2022.945869

**Published:** 2022-08-08

**Authors:** Shuang Tang, Yan Wang, Zhenyu Zong, Ning Ding, Zutai Zhang

**Affiliations:** Beijing Institute of Dental Research, School of Stomatology, Capital Medical University, Beijing, China

**Keywords:** zirconia implant, TiO_2_ coating, UV irradiation, bioactivity, osteogenic activity

## Abstract

Zirconia is a superior implant material owing to its high mechanical strength, durable corrosion resistance, superior aesthetic effect and excellent biocompatibility. However, the bioactivity of zirconia surfaces remains a great challenge for implant osseointegration. A titania (TiO_2_) coating was innovatively synthesized on the surface of zirconia by infiltration in a suspension of zirconium oxychloride and titania for dense sintering. Subsequently, the coating was subjected to ultraviolet (UV) light to enhance the biological inertness of zirconia. Scanning electron microscopy (SEM), X-ray photoelectron spectroscopy (XPS), X-ray diffraction (XRD) and contact angle analysis were conducted to confirm the surface characteristics. Afterwards, *in vitro* assessments of cell adhesion, proliferation and osteogenic differentiation of MC3T3-E1 cells were performed. Zirconia samples were implanted into rat femurs to assess biocompatibility and host tissue response *in vivo*. Micro-CT evaluation and histological testing were conducted. After UV irradiation, the content of hydroxyl groups and hydrophilicity of TiO_2_-modified zirconia were significantly increased. The results of *in vitro* experiments showed that TiO_2_-modified zirconia subjected to UV light could promote cell proliferation and spreading, enhance ALP activity and the degree of mineralization, and upregulate osteogenesis-related genes. Furthermore, *in vivo* assessments confirmed that UV-irradiated TiO_2_-modified zirconia implants maximized the promotion of osseointegration. TiO_2_-modified zirconia after UV treatment will have broad clinical application prospects in improving the osseointegration of zirconia implants.

## 1 Introduction

Dental implantation is the primary treatment option for patients suffering from tooth loss or defects (Kubasiewicz-Ross et al., 2017; Schünemann et al., 2019). Although titanium and titanium alloys are the most extensively applied implant materials, the limitations associated with Ti implants, such as aesthetic defects, allergic reactions and other issues, are attracting more and more attention (Kunrath et al., 2020; Liu et al., 2020). Therefore, the search for novel implant alternative materials is becoming increasingly important. Zirconia ceramic is mainly used as a material for load-bearing implants in dentistry because of its aesthetic effect similar to natural teeth, excellent mechanical properties and reduced inflammation around the implant (Gautam et al., 2016). Thus, zirconia is the most promising candidate due to these superior outcomes. However, the surface bioinertness of zirconia is an urgent problem to be solved (Aminian et al., 2016; Bosshardt et al., 2017).

Several strategies have been employed to enhance the bioactivity of zirconia (Cengiz et al., 2016), such as sandblasting (Moritz et al., 2019), acid etching (Yu et al., 2021) and diverse bioactive coatings comprising silica, zinc and various compounds (Ke et al., 2017). These approaches can create a biologically active surface on zirconia implants. Sandblasting treatment may reduce the mechanical strength of the zirconia substrate (Bacchelli et al., 2009). A suitable etching time and acid solution concentration have not achieved a consistent result (Vu et al., 2018). Various biocoatings have difficulty obtaining the desired effect due to separation from zirconia substrates (Bakhsheshi-Rad et al., 2017; Rohr et al., 2019). After all, seeking an ideal modification method remains a problem.

Titanium and titanium alloys obtain better bioactive surfaces because the surface of titanium implants generates TiO_2_ immediately after they are exposed to oxygen or the atmosphere (Li et al., 2018). TiO_2_ can induce the formation of hydroxyapatite in the biological environment, resulting in bioactive bonding between TiO_2_ and host bone (Wang et al., 2016; Calabrese et al., 2021). According to reports, both anatase and rutile TiO_2_ can generate a certain degree of osseointegration with bone (Kokubo et al., 2007). Ultraviolet (UV)-induced superhydrophilicity of TiO_2_ was discovered in 1997 (Wang et al., 1997). The change in the hydrophilicity of the TiO_2_ surface is attributed to the conversion of its hydrophilic phase, which is described as the oxygen cavitation effect (Zubkov et al., 2005). Superhydrophilic TiO_2_ surfaces for biomaterials have been confirmed to exhibit significantly increased bioactivity (Shimizu et al., 2016; Wang et al., 2021).

Some scholars employed the sol-gel method to prepare the TiO_2_ coating on the zirconia surfaces. In addition, commercial TiO_2_ powders were mixed in zirconia powder during the zirconia processing. However, the sol-gel method was difficult to form strong bonding with zirconia substrates. Mixing of commercial powders was difficult to meet the mechanical properties of implant materials (Xiao et al., 2016; Rbdpm et al., 2018). In this study, TiO_2_ coating was creatively prepared on zirconia by hydrothermal treatment of zirconium oxychloride and titanium oxide mixed suspension to improve the bioactivity of zirconia. The bonding of titania to the substrate was facilitated by the hydrolytic properties of zirconium oxychloride. After dense sintering, the titania coating was firmly bonded to the zirconia substrate. Furthermore, taking advantage of the photocatalytic properties of TiO_2_, UV irradiation was utilized to enhance the osteogenic activity of TiO_2_ coating-modified zirconia. The biological effects of this treatment were systematically evaluated by *in vivo* and *in vitro* experiments.

## 2 Materials and methods

### 2.1 Preparation of zirconia specimens

Pre-sintered zirconia discs (Nissin-Metec, China) with a diameter of 14 mm and a thickness of 2 mm were gradient polished to 1200 mesh and ultrasonically cleaned with distilled water. All zirconia specimens were randomly divided into 2 groups: the control group (C) and the TiO_2_ coating group (TiZ). Group C required no additional treatment. The TiZ group specimens were placed into 1 mol/L ZrOCl_2_ and 0.5 mol/L TiO_2_ mixed suspensions and subsequently heated in a 95°C water bath for 4 h. To prevent evaporation of the liquid, the beaker was covered with a lid. Eventually, the zirconia samples of each group were densely sintered in a furnace (Everest, Kavo, Germany) at a temperature of 1530°C. After dense sintering, half of the TiZ group samples were subjected to UV light (wavelength = 254 nm, irradiance = 100 mW/cm^2^) by a UV irradiation machine (HL-2000 HybriLinker, Japan) and were classified as the UV-TiZ group. The UV-TiZ group samples were placed 10 cm below the lamp tube and irradiated for 15 min. In addition, each group of rod zirconia implants (diameter = 1 mm, length = 10 mm) were prepared in the same way as zirconia discs.

### 2.2 Surface characterization

The surface morphology of each group of zirconia specimens was observed by SEM (Phenom-world, Netherlands). The samples were subjected to XPS (Thermo Fisher Scientific, United States) to evaluate the changes in the chemical state of oxygen on the surface of the zirconia discs after UV treatment. Crystal phase analysis of all samples was performed by XRD examination (SEIFERT, Ahrensburg, Germany). The water contact angle of the specimen (*n* = 4) was evaluated by a contact angle goniometer (Kino Industry, United States). The bonding strength of the titania coating and zirconia (*n* = 5) was measured by using a scratch tester (CSM Instruments, Switzerland). A rockwell head with a radius of curvature of 200 μm was then used with a loading rate of 20 N/min and a terminal load of approximately 100 N. The critical load (Lc) value of the coating is obtained by signal acquisition.

### 2.3 *In vitro* assessments

#### 2.3.1 Cell culture

MC3T3-E1 pre-osteoblasts (ATCC, United States) were cultured to evaluate the cytocompatibility of each zirconia group. Under standard conditions (temperature of 37°C, 95% humidity and 5% carbon dioxide), cells were cultured in a formulated alpha minimum essential medium containing 10% fetal bovine serum and 1% penicillin. The medium was refreshed every 48 h.

#### 2.3.2 Cytotoxicity of zirconia discs

MC3T3-E1 cells were seeded at a density of 1× 10^4^ cells/well in a 24-well plate. At each culture period (1 and 3 d), a live/dead staining kit (Solarbio, China) was utilized to assess the cytotoxicity of zirconia specimens. Calcein-AM was a staining reagent for fluorescently labelling living cells with green fluorescence, and its working concentration was 1 μΜ. In addition, PI (3 μΜ) only stained dead cells and excited red fluorescence. Finally, dyed cells were visualized through fluorescence microscope (Olympus, Japan).

#### 2.3.3 Cell viability assays

A CCK-8 kit (Dojindo, Japan) was used to detect the proliferation of MC3T3-E1 cells cultured on the surface of groups C, TiZ and UV-TiZ. The seeding density was 2.5×10^4^ cells/well in 24-well plates (*n* = 4). After 1, 3 and 5 d of culture, media containing 10% CCK-8 reagent was added to the wells as directed and incubated at 37°C for 2.5 h. Finally, 100 μL of solution was removed from each well into a 96-well plate, and the absorbance was read at 450 nm.

#### 2.3.4 Observation of cell morphology

To visualize the cytoskeleton, MC3T3-E1 cells (1×10^4^ cells/well) were seeded on zirconia discs in a 24-well plate. After culturing for 1 and 3 d, the cells were fixed with 4% paraformaldehyde for 30 min and infiltrated with 0.1% Triton X-100 for 10 min. Thereafter, F-actin and nuclei were stained with phalloidin (Sigma, United States) and DAPI (Beyotime, China), respectively. A fluorescence microscope (Olympus, Japan) was utilized to observe the morphology of the cells cultured on zirconia discs.

In addition, cell morphology was observed by SEM (Phenom World, Netherlands). MC3T3-E1 cells were seeded in a 24-well plate at a density of 1×10^4^ cells/well. After 1 d of incubation, all specimens were incubated overnight at 4 °C in 2.5% glutaraldehyde. The specimens underwent gradient dehydration by a range of ethanol concentrations (30, 50, 75, 85, 95 and 100 v/v%). Finally, all samples were observed after gold spraying.

#### 2.3.5 ALP staining and quantification

MC3T3-E1 cells were incubated in a 24-well plate at a density of 2×10^4^ cells/well (*n* = 4). After culturing for 24 h, the medium was replaced with fresh osteogenic induction medium containing dexamethasone (100 nM, Sigma, United States), β-glycerophosphate (10 mM, Sigma, United States) and L-ascorbic acid (50 mM, Sigma, United States). After 4 and 7 d of osteogenic induction, the total amount of protein was examined by a BCA protein assay kit (Beyotime, China). An ALP assay kit (Nanjing Jiancheng, China) was employed for the quantitative assessment of ALP. Finally, the ALP activity was measured according to the instructions, and it was standardized to the total protein content. Afterwards, at testing time points on 4 and 7 d of induction, the cells were stained with a BCIP/NBT Kit (Beyotime, China) and observed by stereomicroscopy (Olympus, Japan).

#### 2.3.6 Alizarin Red S staining and quantification

ARS and its quantitative results were employed to assess the degree of mineralization of MC3T3-E1 cells seeded on each group of zirconia (*n* = 4) at a density of 1×10^5^ cells/well. After 7 and 14 d of osteogenic induction, the cultured cells were dyed with the prepared 0.2% Alizarin Red staining solution (pH = 4.2, Sigma, United States) and observed by microscopy (Olympus, Japan). Finally, the mineralized nodules were dissolved in 10% cetylpyridinium chloride (Sigma, United States), and the absorbance was read at 620 nm.

#### 2.3.7 Osteogenesis-related gene expression

MC3T3-E1 cells were seeded in a 24-well plate at a density of 1×10^5^ cells/well on the zirconia disc in each well (n = 4). After 7 d of osteogenic induction, the expression levels of osteogenic genes, including ALP, runt-related transcription factor 2 (Runx2), collagen-I (COL-I), osteocalcin (OCN) and osteopontin (OPN), were detected. Total RNA was isolated by the TRIzol (Sigma, United States) method, and the concentration was determined by Nanodrop (Thermo Fisher, United States). The extracted RNA was prepared into cDNA by employing the Reverse Transcription Takara kit (Takara, Japan). Finally, quantitative RT–PCR was performed with SYBR Green chemistry (Takara, Japan). Primers for osteogenesis-related genes are listed in Table 1, and mRNA levels were normalized to GAPDH as a housekeeping gene.

**TABLE 1:** Primers for target genes.

Target genes	Primers
ALP	F: 5′- CTG​CCT​GAA​ACA​GAA​AGT​CTG​C-3′
	R: 5′-TAT​GTC​TTT​ACC​AGG​AGG​CGT​G-3′
Runx2	F: 5′-ATC​CAG​CCA​CCT​TCA​CTT​ACA​CC-3′
	R: 5′-GGG​ACC​ATT​GGG​AAC​TGA​TAG​G-3′
COL-1	F: 5′-CCT​GAG​CCA​GCA​GAT​TGA-3′
	R: 5′-TCC​GCT​CTT​CCA​GTC​AG-3′
OCN	F:5′-AGACTCCGGCGCTACCTT-3′
	R:5′-CTCGTCACAAGCAGGGTTAAG-3′
OPN	F: 5′-TTC​TCC​TGG​CTG​AAT​TCT​GAG​G-3′
	R: 5′-GCT​GCC​AGA​ATC​AGT​CAC​TTT​C -3′
GAPDH	F: 5′- ATG​GGT​GTG​AAC​CAC​GAG​A-3′
	R: 5′-CAG​GGA​TGA​TGT​TCT​GGG​CA-3′

### 2.4 *In vivo* animal experiments

#### 2.4.1 Surgical procedures

All animal experiments were approved by the ethics committee of Beijing Stomatological Hospital affiliated with Capital Medical University and complied with the “Guide for the Care and Use of Laboratory Animals”. A total of 78 Eight-week-old male Sprague Dawley (SD) rats were randomly divided into 3 groups: C, TiZ and UV-TiZ. After general anaesthesia by intraperitoneal injection of chloral hydrate, the legs of each rat were shaved and sterilized. An incision was made in the rat’s leg to expose the femur. A bone defect of 1 mm in diameter and 10 mm in length was prepared and cooled with saline to prevent osteonecrosis. Afterwards, each group of zirconia implants prepared according to the method described in 2.1 were placed into the cavity, and the wound was carefully sutured. After 4 and 8 w of healing, the rats were sacrificed. The implant-containing femur specimens were fixed with 4% paraformaldehyde for further evaluation.

#### 2.4.2 Micro CT analysis

High-resolution micro-CT (Skyscan, Bruker) was employed to scan the obtained specimens. The scanning parameters were set as follows: the spatial resolution was 18 μm (500 projections/180°, 1 mm aluminium filter, 100 kV, 100 mA). Three-dimensional (3D) images of each group of samples were reconstructed by CTvox (Skyscan, Bruker) software. Reconstructed data were further analyzed by CT-Analyzer through controlling the minimum grey threshold value of 30 and the maximum of 255. The region of interest (ROI) was determined as a column (1.5 mm in diameter) from the centre of the implant and 1.0 mm above the epiphyseal growth layer line.50 axial images were reconstructed into a 3D image which was used to measure the bone parameters. The important bone parameters, including new bone volume over total bone volume (BV/TV), mean trabecular thickness (Tb.Th), trabecular number (Tb.N) and trabecular separation (Tb.Sp), were calculated (n = 4).

#### 2.4.3 Mechanical push-out examination

The integration of the implant and bone tissue was tested by a universal testing machine (Shimadzu, Japan). The femoral samples (*n* = 5) were carefully trimmed to expose the top of the implant. Thereafter, the cut bone tissue was fixed on the jig and clamped in the same position on the different implants. The implant was pulled out at a speed of 1 mm/min, and the maximum force was recorded.

#### 2.4.4 Histological analysis

After the SD rats were sacrificed, the bone fragments were fixed with 4% paraformaldehyde for 48 h. Subsequently, the bone blocks were soaked in graded ethanol solutions (60 increased to 100 v/v%). After dehydration, the tissue blocks were placed in plastic moulds and embedded in an EXAKT 520 light-curing embedding machine using Technovit 7200 resin (KULZER, Germany). The diamond blade cut the specimen along the long axis of the implant to obtain a 200-μm-thick tissue piece. Thereafter, the sections were graded and polished to 20 μm. Finally, the samples were stained with methylene blue and fuchsin staining reagent and observed by microscopy (Olympus, Japan). The percentage of bone-implant contact (BIC%) was measured by image pro plus software (*n* = 4).

### 2.5 Statistical analysis

All statistical data are expressed as the means with standard deviations. LSD analysis was used to compare pairs after one-way analysis of variance (ANOVA). Statistical significance was defined as *p < 0.05, **p < 0.01, and ***p < 0.001. A preliminary statistical power analysis, calculated using PASS Software (NCSS, Kaysville, United States), was used to determine the sample size. Based on the data from our pilot study, the sample size was identified to provide statistically significant measure of the various tests used in this study (power = 0.9, α = 0.05).

## 3 Results

### 3.1 Surface characterization

**FIGURE 1:**
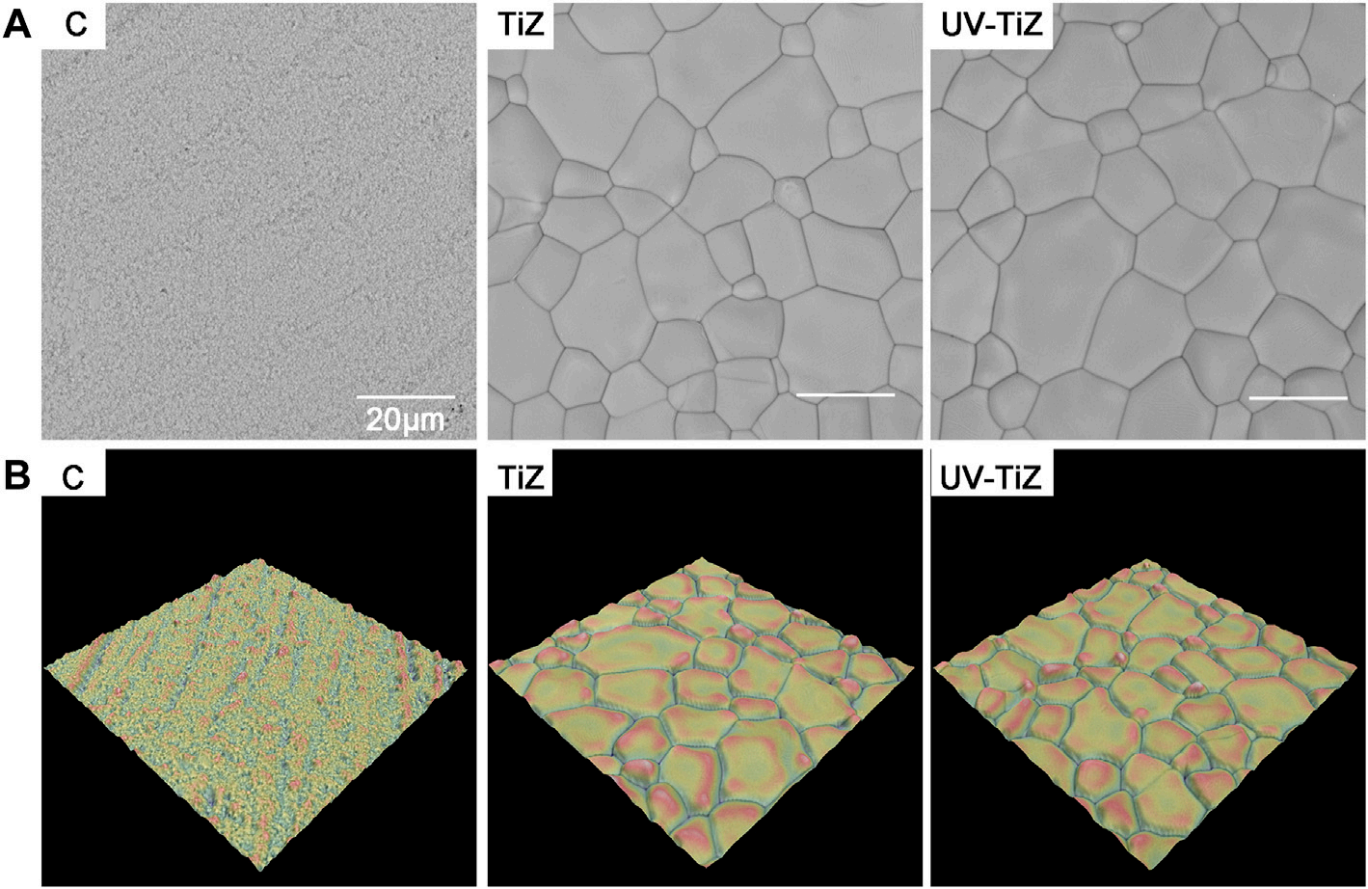
**(A)** SEM images of each group of samples (× 5000); **(B)** 3-D reconstruction topographical images of groups C, TiZ and UV-TiZ.

Figure 1A shows the surface topography of each group samples. After modification, a uniform TiO_2_ coating was prepared on the surface of zirconia. Figure 1B exhibits the three-dimensional (3-D) reconstruction images of each group. The surface of group C was relatively smooth, while the TiO_2_ coatings were rough.

**FIGURE 2:**
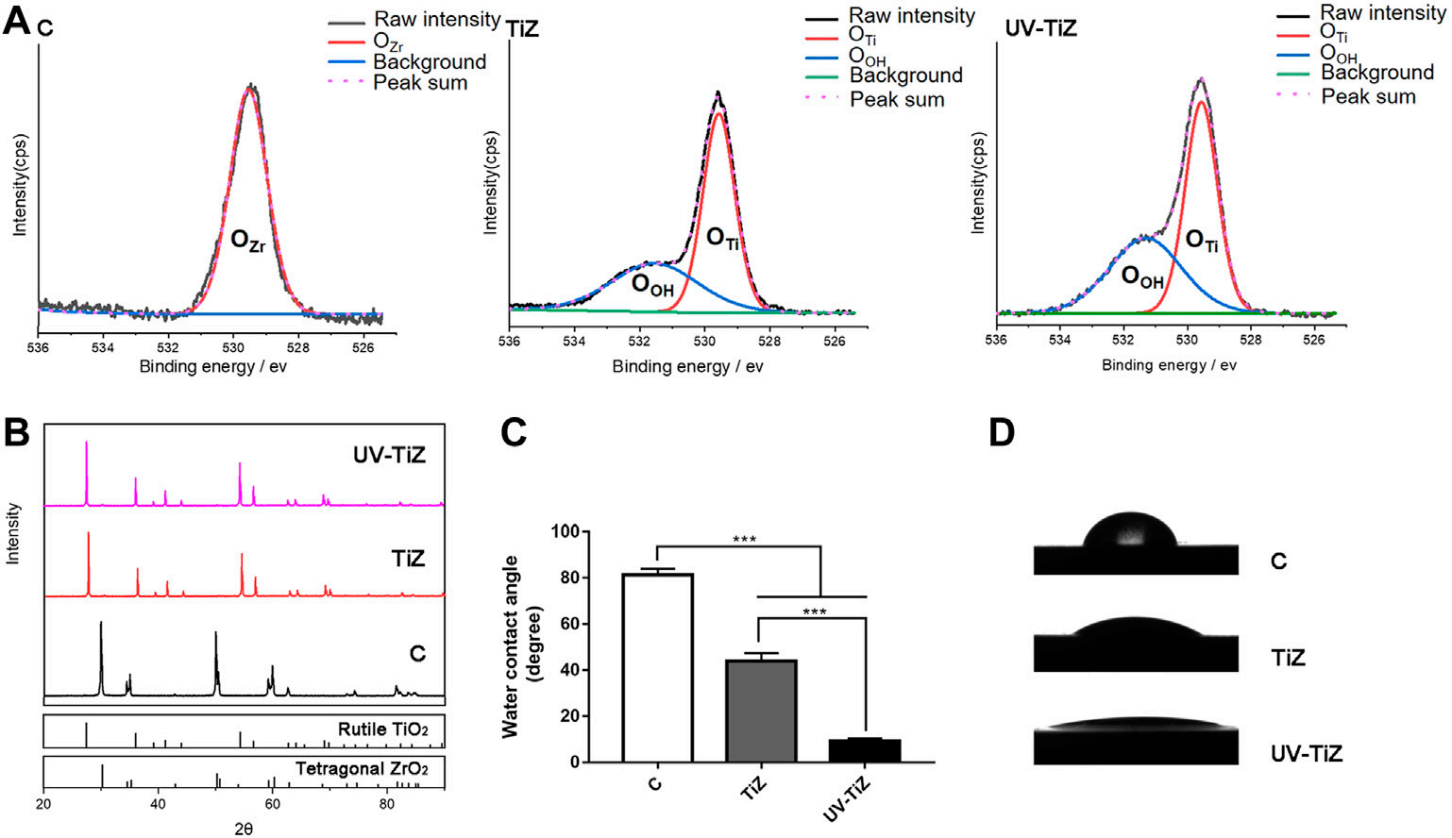
**(A)** Comparison of the XPS spectra of O1s of the different treatment groups. **(B)** XRD patterns of groups C, TiZ and UV-TiZ. The JCPDS numbers of different crystal phases are marked. The contact angle measurements of different specimens are exhibited in **(C)** and **(D)**. *p < 0.05, **p < 0.01, ***p < 0.001.

The high-resolution O1s peaks of the XPS spectrum of the TiZ and UV-TiZ groups could be separated into two peaks at approximately 530.0 and 531.1 eV (Figure 2A), and they were attributed to Ti-O and Ti-OH groups, respectively [(Reyes-Gil et al., 2007), (Chen et al., 2011)]. The percentage of Ti-OH species increased from 18.26% to 55.85% after UV irradiation, which indicated that more Ti-OH bonds were generated on the surface of the UV-TiZ group than that of the TiZ group. However, the O1s peak of group C originating from the Zr-O group was only approximately 530.0 eV (Choi et al., 2019). Through XRD detection, it was confirmed that rutile TiO_2_ was prepared on the surface of zirconia, and crystal phase transformation did not occur after UV irradiation (Figure 2B). The contact angles of each group of specimens are exhibited in Figures 2C,D, and the differences among groups were significant (*p* < 0.05). The contact angle of zirconia (81.35°) was significantly higher than that of the TiO_2_ coating (43.95°). Moreover, after UV irradiation, the hydrophilicity of the TiO_2_ coating was significantly improved to 9.33°. The excellent adhesive strength of the coating plays a key role in its mechanical properties. The maximum force on the titania coating reached (20.3 ± 1.2) N. The titania coating had a high enough bond strength to withstand the load.

### 3.2 *In vitro* evaluation

#### 3.2.1 Cytocompatibility assessment of zirconia samples

**FIGURE 3:**
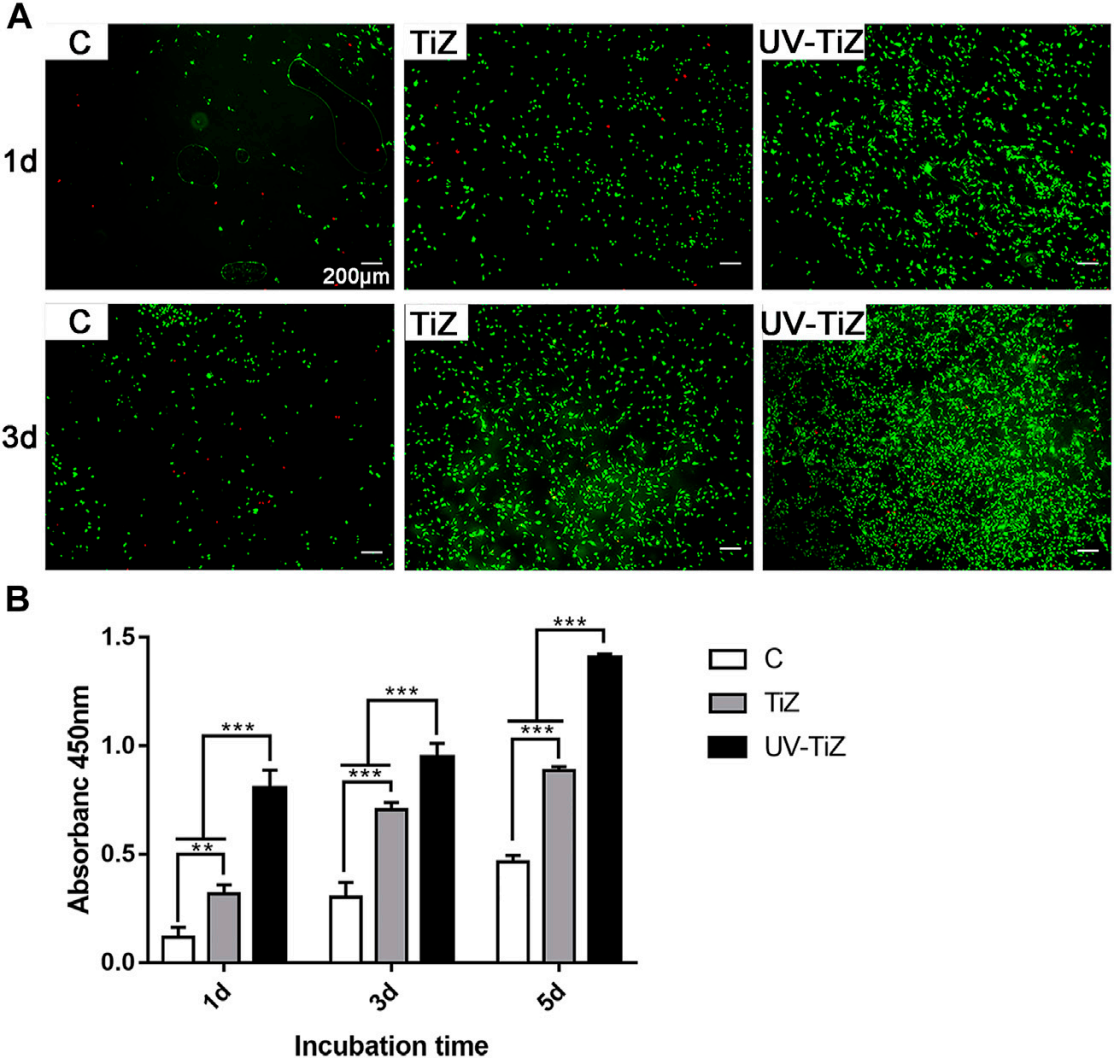
**(A)** Live/dead double staining of MC3T3-E1 cells seeded in the C, TiZ and UV-TiZ groups after 1 and 3 d. **(B)** Proliferation of MC3T3-E1 cells cultured on different specimens was detected by CCK-8 for 1, 3 and 5 d *p < 0.05, **p < 0.01, ***p < 0.001.

After live/dead cell staining, live cells were dyed green with calcein-AM, and dead cells presented red due to propidium iodide. Figure 3A shows that only a few dead cells stained with red fluorescence were observed. At both the 1 and 3 d testing time points, MC3T3-E1 cells inoculated in the TiZ and UV-TiZ groups displayed more green fluorescence than those in the C group. Moreover, the UV-TiZ group exhibited the highest distribution density of green fluorescence. The fluorescence microscopy images revealed that each group of zirconia was not cytotoxic.

Figure3B displays the proliferation of MC3T3-E1 cells seeded on each group of zirconia discs. At each testing time, the proliferation of cells showed the same trend: UV-TiZ > TiZ > C. In addition, the differences among groups were significant (*p* < 0.05).

#### 3.2.2 Cell morphology observation

**FIGURE 4:**
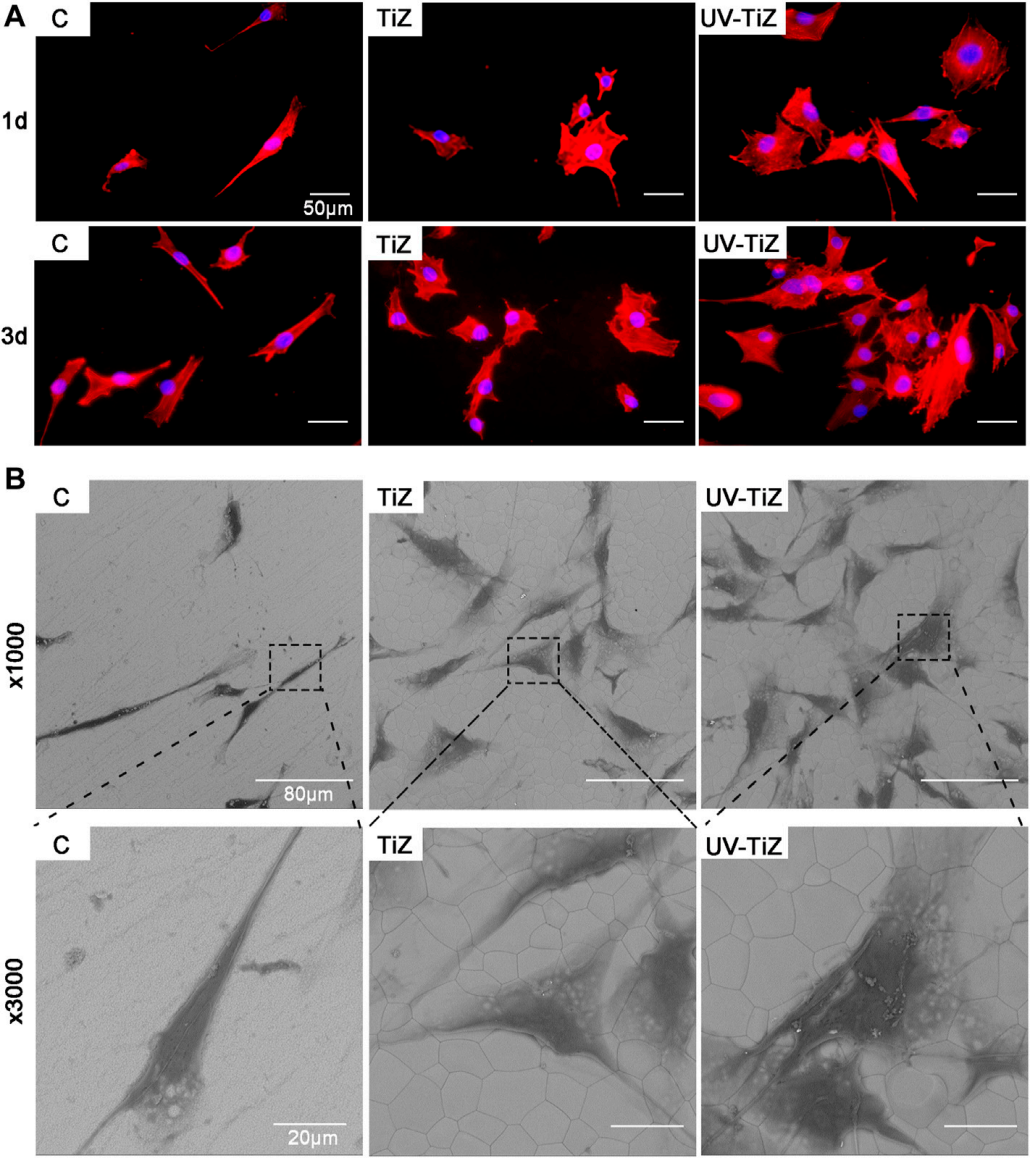
**(A)** Cytoskeletal morphology of MC3T3-E1 cells incubated on different specimens for 12 and 24 h **(B)** SEM morphology of MC3T3-E1 cells seeded in groups C, TiZ and UV-TiZ after 24 h.

Figure 4A shows that the cells seeded on the surface of each group of samples showed different morphologies. The cells cultured in group C mainly exhibited a spindle shape; however, the cells seeded in the TiZ and UV-TiZ groups appeared round. Moreover, the cells adhered to the surface of UV-TiZ group zirconia discs and stretched out more filopodia.

The SEM images of MC3T3-E1 cells seeded on different groups were displayed in Figure 4B. After culturing for 1 d, compared with group C, more cells adhered to the surface of groups TiZ and UV-TiZ. High magnification photomicrographs of MC3T3-E1 cells incubated on TiZ and UV-TiZ surfaces exhibited a wider spreading area compared to group C. Notably, the cells cultured on the surface of the UV-TiZ group protruded the most pseudopodia.

#### 3.2.3 Cell differentiation

**FIGURE 5:**
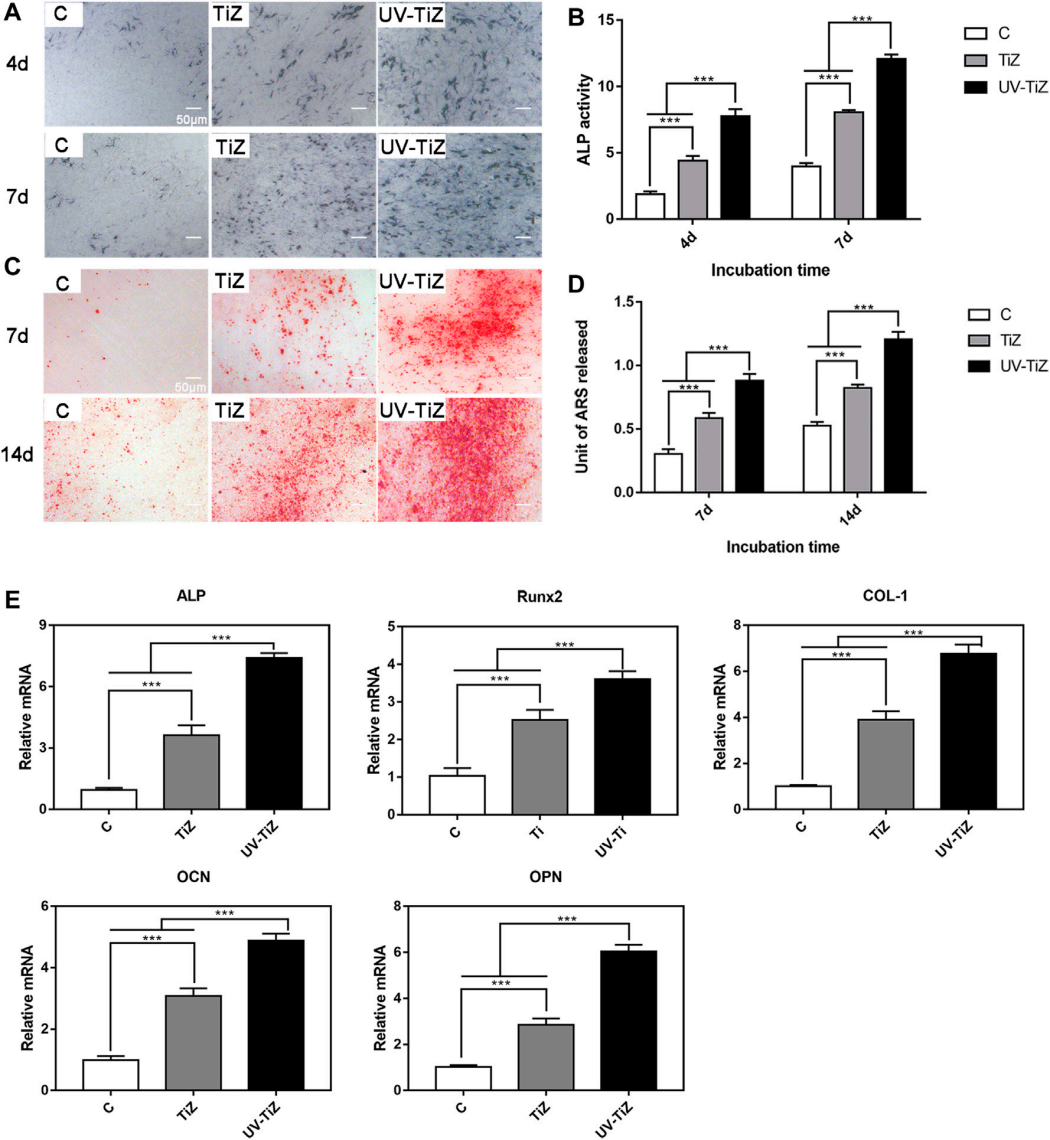
**(A)** and **(B)** ALP staining and quantitative results of MC3T3-E1 cells incubated on the various samples for 4 and 7 d of osteogenic induction. **(C)** and **(D)** Alizarin red staining and quantitative results of the cells seeded on zirconia discs at 7 and 14 d after culturing in osteogenic medium. **(E)** mRNA levels of osteogenic differentiation-related genes, including ALP, Runx2, COL-1, OCN and OPN, in MC3T3-E1 cells cultured in the C, TiZ and UV-TiZ groups at 7 d after seeding in osteogenic medium. *p < 0.05, **p < 0.01, ***p < 0.001.

Figures 5A,B present the ALP staining and quantitative results of each group. After 4 and 7 d of osteogenic induction, the MC3T3-E1 cells cultured on UV-TiZ zirconia exhibited the highest ALP activity. Meanwhile, the cells incubated on TiZ specimens showed higher ALP activity than those in group C. In addition, the differences among the groups were significant (*p* < 0.05).

After 7 and 14 d of osteogenic induction, MC3T3-E1 cells seeded on the surface of the UV-TiZ group exhibited the most mineralized nodules and the highest mineralization level (shown in Figures 5C,D). Furthermore, compared with group C, the cells cultured in the TiZ group appeared a higher degree of mineralization. The differences between the groups were significant (*p* < 0.05).

The RT–PCR analysis of ALP, Runx2, COL-І, OPN and OCN gene expression is shown in Figure 5E. After 7 d of osteogenic induction, the expression of osteogenesis-related genes in the cells inoculated on the surface of the TiZ and UV-TiZ groups was significantly upregulated compared with that in group C (*p* < 0.05). The degree of gene expression upregulation showed a trend as follows: UV-TiZ > TiZ > C. In addition, the differences between the groups were significant (*p* < 0.05).

### 3.3 *In vivo* assessments

#### 3.3.1 Micro-CT analysis

**FIGURE 6:**
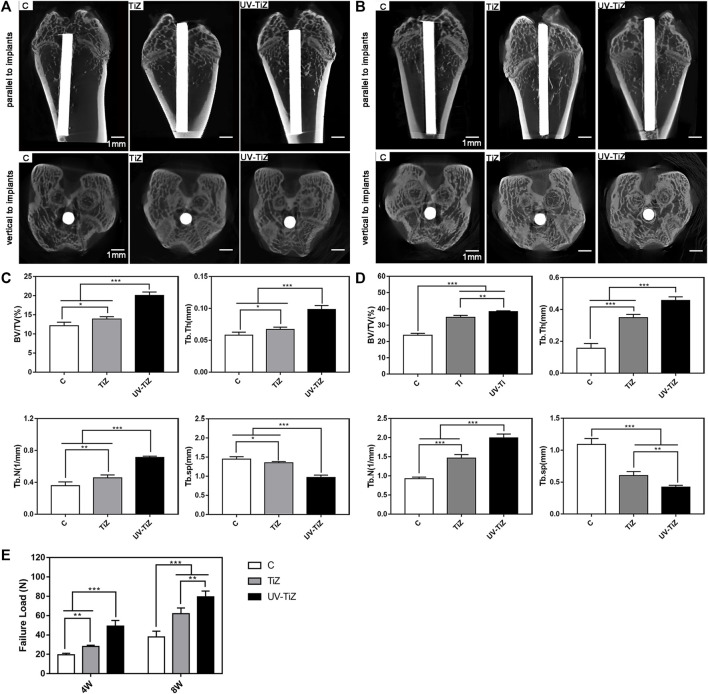
Representative 3D-reconstructed micro-CT images of the bone around each group of implants after 4 w **(A)** and 8 w **(B)**. Quantitative statistics of BV/TV, Tb.Th, Tb.N and Tb.Sp according to the micro-CT images after 4 w **(C)** and 8 w **(D)**. **(E)** The maximum pull-out force of different implants at 4 and 8 w postoperatively. *p < 0.05, **p < 0.01, ***p < 0.001.

Micro-CT was employed to evaluate the new bone formation around different implants after 4 and 8 w of healing, and the results are presented in Figures 6A,B. The reconstruction images of bone tissue were obtained in two directions (parallel and vertical to the implant). After 4 w of implantation, group C presented a larger bone defect around it. The bone defect around TiZ was smaller, and the osteogenic effect was better than that of group C. UV-TIZ showed obvious bone repair around the implant, and the osteogenic effect was superior than TiZ and C. After 8 w, new bones were formed in the defective position around the implants. Uniform and continuous new bone around the UV-TiZ implant almost completely healed the bone defect. Compared with group C, superior bone regeneration was observed near the margin of the defect of the TiZ and UV-TiZ groups. However, the most ideal new bone formation was obtained around the implants of the UV-TiZ group.

Figures 6C,D show the statistical results of a detailed analysis of bone tissue around each group of implants after 4 and 8w, respectively. The newly formed bone around the UV-TiZ group implant exhibited the highest level of BV/TV, Tb.Th, and Tb.N and the smallest Tb.Sp. In addition, the TiZ group was second, and group C was the worst. Figure 6E also exhibits the maximum pull-out force of each group of implants. The maximum pull-out force in each group increased gradually with the prolongation of time. The failure load of the UV-TiZ group was significantly higher than that of the other two groups (*p* < 0.05), and that of the TiZ group was also significantly higher than that of group C (*p* < 0.05).

#### 3.3.2 Histological evaluation

**FIGURE 7:**
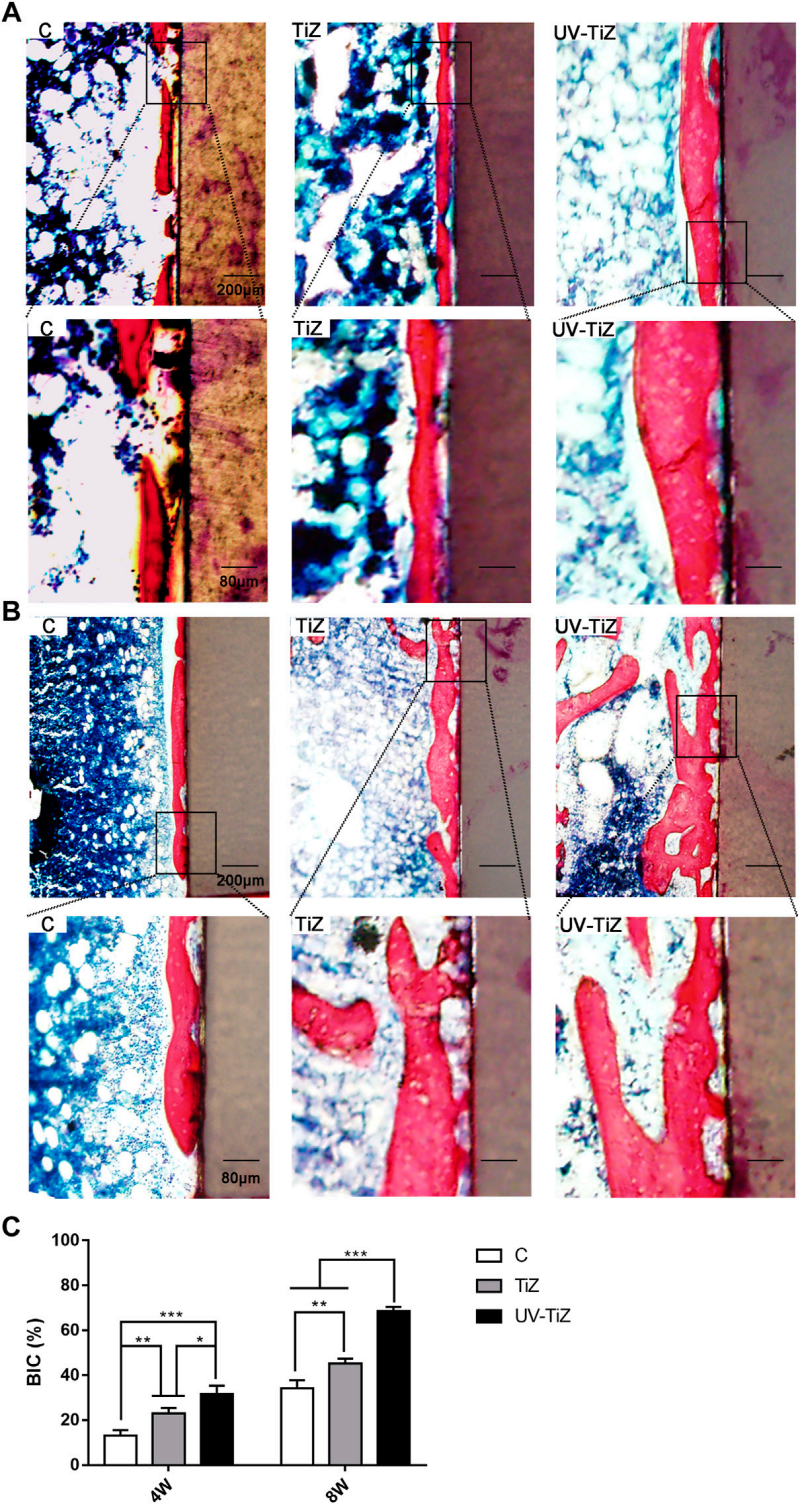
Histological analysis of peri-implant new bone formation by methylene blue fuchsin staining after 4 w **(A)** and 8 w **(B)**. **(C)** The percentage of the bone-implant contact (BIC) of different groups after 4 and 8 w. *p < 0.05, **p < 0.01, ***p < 0.001.

Methylene blue and fuchsin staining was employed to assess the osseointegration of the implants in each group. Figures 7A,B display methylene blue and fuchsin staining images of the three groups. Newly formed calcified bone was stained red, and blue staining represented osteoblasts or osteoid tissue. At 4 w postoperatively, in group C, red-stained newly formed bone was not abundant with little direct contact with the implant. In the TiZ group, new bone was continuous. In the UV-TiZ group, continuous new bone formed and generated direct contact with the implant. After 8 w, compared with the TiZ and C groups, more dense new bone tissue was observed around the UV-TiZ implants. In addition, there was more red staining around the TiZ implants than in group C. Figure 7C shows that the BIC% of UV-Ti was the highest at either 4 or 8 w. It is further demonstrated that UV irradiation of titania coating may induce bone formation.

## 4 Discussion

After extensive long-term research, zirconia has been confirmed to be suitable for application in dental implants (Kunrath et al., 2021). A number of surface modification methods have been attempted to enhance the bioactivity of zirconia implants (Rohr et al., 2020). In this study, pre-sintered zirconia was subjected to water bath treatment and dense sintering to obtain TiO_2_-coated modified zirconia. The preparation of TiO_2_ coating took advantage of the hydrolysis of zirconium oxychloride and the large porosity of pre-sintered zirconia. Consequently, TiO_2_ coatings were strongly bonded to the substrate without damaging the mechanical strength of the zirconia (Tang et al., 2021). To enhance the osteogenic activity of TiO_2_ coating-modified zirconia, UV treatment was introduced to achieve a more ideal osseointegration effect.

The successful loading of dental implants is determined by their physicochemical surface properties. The XPS results demonstrated that a large number of Ti-OH bonds were excited on TiO_2_-modified zirconia surface after UV irradiation, which caused an increase in hydrophilicity. These physicochemical changes were mainly related to UV-induced direct photolysis of hydrocarbons and reactive oxygen species generation on the TiO_2_ surface. Under ultraviolet light irradiation, electrons in the valence band of TiO_2_ are excited to the conduction band, electrons and holes migrate to the surface of TiO_2_ and electron-hole pairs are generated on the surface. The electrons react with Ti, and the holes react with the surface bridge oxygen ions, forming respectively positive trivalent titanium ions and oxygen vacancies. In turn, the oxygen and water in the environment were converted into various free radicals, including superoxide and hydroxyl groups, to increase the polarity and finally resulted in an increase in hydrophilicity (Choi et al., 2016). Hydrophilicity is a key factor in controlling cellular responses to biomaterials (Lang et al., 2011).

To explore the effect of the UV-irradiated TiO_2_-modified zirconia surface on bone tissue, the behaviour and response of MC3T3-E1 cells seeded on the surface of zirconia specimens in each group were evaluated by *in vitro* studies. After UV irradiation, the seeded cells were not damaged and exhibited better proliferation activity. UV-TiZ group zirconia could best promote MC3T3-E1 cell adhesion and spreading. Early cell adhesion is important for subsequent osteogenic differentiation, utilizing implant materials to recruit cells and initiate osseointegration [(Qahtani et al., 2017), (Li et al., 2015)].

The effect of TiO_2_-modified zirconia on the osteogenic differentiation of MC3T3-EI cells after UV irradiation was examined by an ALP activity test and mineralization assessment. ALP is one of the markers of early osteogenic differentiation (Fraioli et al., 2016). The degree of mineralization is known as an important reference for the late stage of osteogenic differentiation (Ocana et al., 2017). The UV-TiZ group displayed the highest ALP activity and mineralization levels and upregulated osteogenic gene expression compared with the other two groups. The promotion of UV irradiation on the osteogenic activity of TiO_2_-modified zirconia was further confirmed. Superhydrophilic TiO_2_ surfaces of biomaterials and metal implants have been demonstrated to increase bioactivity and promote osteogenic differentiation (Aita et al., 2009). Notably, UV-induced superhydrophilic TiO_2_ surfaces can increase the recruitment, spreading, and proliferation of osteoblasts, which in turn enhances integration between bone tissue and implants (Ueno et al., 2010).

Histological analysis is the criterion for evaluating implant osseointegration (Calvo-Guirado et al., 2019). Meanwhile, it is necessary to perform *in vivo* experiments to confirm whether the *in vitro* results are applicable to actual clinical applications. The *in vivo* results confirmed that the UV-TiZ group exhibited the best osteogenic effect. UV-functionalized dental implants are utilized clinically due to their therapeutic advantages (Suzuki et al., 2009). In some studies of biomaterials, the effect of surface hydrophilicity on the bioactivity of materials is still debated (Elias et al., 2008). Although hydrophilicity has a positive role in UV-induced osseointegration, it seems difficult to achieve such a satisfactory effect in the presence of hydrophilicity alone. Ultraviolet (UV) treatment for 15 min prior to implantation can significantly eliminate surface hydrocarbons, increase hydrophilicity, and effectively promote new bone formation and long-term rehabilitation effects (Choi et al., 2017). The possible mechanism is the decomposition and elimination of organic components via UV-mediated photocatalysis, followed by the induction of superhydrophilicity and enhanced surface energy. In this study, the TiO_2_ coatings appeared to be more osteoconductive after UV treatment. In further studies, we will explore the effect of UV irradiation on the formation of biofilms on TiO_2_-modified zirconia surfaces, which are mainly due to the presence of contaminants on material surface.

## 5 Conclusion

In this study, the UV-treated TiO_2_-modified zirconia surface significantly promoted the proliferation, spreading and differentiation of seeded MC3T3-E1 cells. The *in vivo* evaluation demonstrated that UV-irradiated TiO_2_ coatings could induce more bone tissue formation around zirconia implants. Above all, the *in vitro* and *in vivo* analyses suggested that UV-irradiated TiO_2_-coated zirconia could improve osseointegration and will be a promising biomaterial for clinical applications.

## Data Availability

The original contributions presented in the study are included in the article/supplementary material, further inquiries can be directed to the corresponding author.
